# Employers With Metabolic Syndrome and Increased Depression/Anxiety Severity Profit Most From Structured Exercise Intervention for Work Ability and Quality of Life.

**DOI:** 10.3389/fpsyt.2020.00562

**Published:** 2020-06-18

**Authors:** Sven Haufe, Kai G. Kahl, Arno Kerling, Gudrun Protte, Pauline Bayerle, Hedwig T. Stenner, Simone Rolff, Thorben Sundermeier, Julian Eigendorf, Momme Kück, Alexander A. Hanke, Katriona Keller-Varady, Ralf Ensslen, Lars Nachbar, Dirk Lauenstein, Dietmar Böthig, Christoph Terkamp, Meike Stiesch, Denise Hilfiker-Kleiner, Axel Haverich, Uwe Tegtbur

**Affiliations:** ^1^Institute of Sports Medicine, Hannover Medical School, Hannover, Germany; ^2^Department of Psychiatry, Social Psychiatry and Psychotherapy, Hannover Medical School, Hannover, Germany; ^3^Volkswagen AG, Wolfsburg, Germany; ^4^Audi BKK Health Insurance, Ingolstadt, Germany; ^5^Department of Cardiac, Thoracic, Transplantation, and Vascular Surgery, Hannover Medical School, Hannover, Germany; ^6^Department of Gastroenterology, Hepatology and Endocrinology, Hannover Medical School, Hannover, Germany; ^7^Department of Prosthetic Dentistry and Biomedical Materials Science, Hannover Medical School, Hannover, Germany; ^8^Department of Cardiology and Angiology, Hannover Medical School, Hannover, Germany

**Keywords:** physical activity, telemonitoring, activity devices, productivity, mental health

## Abstract

**Background:**

Major depressive disorder and anxiety disorders are associated with less productivity, earlier retirement, and more sick-days at the workplace. These associations also exist for patients with metabolic syndrome. For both, exercise is a generally recommended part of multimodal treatments. However, for individuals with metabolic syndrome, in which depression and anxiety is more prevalent and severe, evidence for the efficacy of exercise interventions is limited.

**Methods:**

Company employees with diagnosed metabolic syndrome (n=314, age: 48 ± 8 yrs) were randomized to a 6-month exercise intervention (150 min per week) or wait-list control. Participants received individual recommendations for exercise activities by personal meetings, telephone, or *via* a smartphone app. Physical activities were supervised and adapted using activity monitor data transferred to a central database. Work ability (work ability index), depression severity and anxiety severity [hospital anxiety and depression scale (HADS)], and health-related quality of live [short form 36 (SF-36)] were assessed.

**Results:**

We included 314 subjects from which 287 finished the intervention. Total work ability, depression- and anxiety severity, and the mental component score of the SF-36 improved after 6 months exercise compared to controls. After baseline stratification for normal (HADS scores 0–7) and increased depression- and anxiety scores (HADS scores 8–21) individuals with increased severity scores had similar age, body composition, blood lipids, and cardiorespiratory fitness compared to those with normal scores, but lower total work ability and component sum scores of health-related quality of life. After 6 months total work ability increased in the exercise group compared to controls with the magnitude of the observed increase being significantly greater for subjects with increased depression- and anxiety severity at baseline compared to those with normal severity scores.

**Conclusions:**

A 6-month exercise intervention for company employees with metabolic syndrome showed strongest effects on self-perceived work ability in individuals with mild to severe depression- and anxiety severity. This suggests exercise programs offered to workers with metabolic syndrome not only reduces individual disease risk but may also reduce healthcare and employers costs arising from metabolic syndrome and mental disease conditions.

**Clinical Trial Registration:**

ClinicalTrials.gov, identifier NCT03293264.

## Introduction

According to the Global Burden of Disease Study, major depressive disorder and anxiety disorders belong to the most frequent and burdensome disorders worldwide leading to premature mortality (1, 2). A recent representative community survey in Germany revealed 12-month prevalence rates as high as 15.3% for anxiety disorders and 7.7% for major depressive disorder (3). Major depressive disorder represents a significant economic burden estimated at 210 billion dollar, with presenteeism and absenteeism accounting for almost half of these costs (4). Current guidelines recommend psychopharmacological drugs and/or psychotherapy for the first line treatment of major depressive disorder and anxiety disorders (5).

A non-pharmacological intervention to ameliorate the severity of depression and anxiety is structured and regular exercise (6). Benefits of physical activity for the treatment and prevention of mental health have been shown for certain (7–9) but not for all subjects or interventions (10–12). Of note, several studies point to an association between major depressive disorder and metabolic syndrome (13, 14). However, for individuals with metabolic syndrome, in which depression and anxiety is more prevalent and severe (15, 16), evidence for the efficacy of exercise interventions is scarce. Metabolic syndrome, a cluster of different cardio-metabolic abnormalities affects about one third of today’s population worldwide (17), with highest and still increasing numbers in the USA (18) and increased prevalence in older populations (19). Both, mental disorders and metabolic syndrome have socioeconomic relevance by affecting workers absenteeism and productivity (20), thereby increasing costs for employers and healthcare systems (13, 14, 21). Physical activity programs improve several cardio-metabolic outcomes in metabolic syndrome patients (22). For depressive patients we observed benefits for abdominal- and cardiac adipose tissues, skeletal muscle mass, aerobic capacity, and brain derived neurotrophic factor following structured exercise interventions (23–26). Recently, we also reported that self-perceived work ability and quality of life improved after a 6-month exercise-focused occupational health program in workers with metabolic syndrome which may be, at least partly, attributable to the effects of exercise on depression and anxiety severity (27). However, to date it is unclear to which extent individuals with higher anxiety and/or depressive symptom load take profit from exercise programs, particularly concerning indicators of work ability. Therefore, we re-analyzed questionnaires and cardio-metabolic data from the 6-month, telemonitoring-supported and exercise-focused lifestyle intervention in employees diagnosed with metabolic syndrome.

## Materials and Methods

### Study Design and Participants

The present study is a secondary analysis of a recently published trial reporting the primary outcome metabolic syndrome severity after exercise training (27). This was a prospective, randomized, and single-blind (assessor blind) trial conducted as a collaborative project between Volkswagen AG and Hannover Medical School (ClinicalTrials.gov Identifier: NCT03293264). The study was carried out in accordance with the Declaration of Helsinki and current guidelines of good clinical practice. The institutional review board of Hannover Medical School approved the study (No. 7531) and written informed consent was obtained prior to inclusion of study participants.

Recruitment of volunteers was conducted at the main Volkswagen factory in Wolfsburg (Lower Saxony, Germany) by local information events during working hours as well as distribution of advertisements *via* email and intranet to employees. According to pre-study defined inclusion and exclusion criteria, we included female and male subjects over the age of 18 who fulfilled at least three of the five metabolic syndrome components according to the American Heart Association (AHA)/The National Heart, Lung, and Blood Institute (NHLBI) criteria, (28) and who were not participating in an ongoing occupational health program. Exclusion criteria were acute or chronic infections, oncological diseases, joint replacements or any surgery within the last 6 weeks, pregnant or breastfeeding women, and any condition that precluded participation in an exercise intervention.

Volunteers were randomized 1:1 to 6-months exercise (EG) or a waiting-control group (CG) using a computer-based list of random numbers generated by an external collaborator. Variable block length was used, to avoid selection bias due to predictability. Study nurses and physicians screening volunteers and assessing the primary outcome at baseline and after 6 months were blinded for the randomization sequence.

### Anthropometric and Cardio-Metabolic Assessments

After a general medical examination by a physician (including ECG, case history, and physical examination), body weight, waist circumference, and height were measured in a standardized fashion. Fat-free and fat mass as markers of body composition were estimated by segmental, multi-frequency bio-impedance analysis (InBody720, Biospace, Seoul, Korea). After an overnight fast, venous blood samples were obtained to determine blood lipids, fasting glucose, and a safety blood profile using standard methods in a certified clinical chemistry laboratory. Office blood pressure was measured after 5 min rest with an appropriate-sized automatic blood pressure cuff (Critikon, Dynamap, Tampa, USA) as a mean of two consecutive records. To test exercise capacity (measured as peak power output in watt), participants performed an incremental exercise test on a bicycle ergometer (Schiller 911 BPplus, Feldkirchen, Germany). The test started with a work load of 50 watts (W) for males and 20 W for females and was increased in 10 W steps each minute until the subjects could not maintain the requested 60 rpm pedal frequency (voluntary exhaustion) or the test was prematurely stopped by the physician due to predefined stopping criteria (29). All assessments were repeated after the 6-month exercise intervention and control period, respectively.

### Questionnaires

We distributed questionnaires for the estimation of anxiety severity and depression severity (HADS) (30), health-related quality of life [short form 36 (SF-36)] (31), as well as for daily physical activity (Freiburger Physical Activity questionnaire) and work ability [work ability index (WAI)] (32). The HADS consists of fourteen items pertaining to the two subscales for anxiety and depression. Scores for the anxiety and depression subscale range from 0 to 21, with higher score indicating more severe anxiety or depression. Values can be interpreted as normal from 0–7 points, mild (8–10 points), moderate (11–14 points), and severe (15–21 points). The SF-36 questionnaire measures health-related quality of life with eight subscales resulting in two sum scales, the mental- and physical component score. For both scales, a score of 0 points represents a minimum and a score of 100 points a maximum quality of life. The Freiburger activity questionnaire determines the total and exercise-related physical activity of adults, both of which are specified as metabolic equivalents of task (MET)-hours per week. The Work Ability Index (WAI) questionnaire contains seven questions concerning work, work ability, and health, resulting in a total score ranging from seven to 49, with higher values representing greater work ability.

### Study Intervention

The study intervention was described in detail elsewhere (27). In brief, participants performing the 6-month exercise intervention received a personal counselling with recommendations aiming to perform 150 min of moderate-intense physical activity per week. For typical physical activities, an individual heart rate with a target range between 65 to 75% relative to measured maximum heart rate was advised, based on data from initial exercise tests, activity questionnaires, and medical history. The exercise scientist provided information on potential exercise training facilities in the vicinity of the participant’s home and at the workplace (e.g. gyms, sport classes, rehabilitation courses) and gave tips to attain a high level of physical activity in daily routine.

The exercise group was equipped with an activity monitor (Forerunner 35, Garmin, Garching, Germany) and asked to wear the monitor throughout the intervention period. Wearing time, steps and preset or self-defined activities (e.g. cycling, cardio-training, walking outdoors and walking indoors) were recorded. Activity data were saved and directly forwarded *via* an interface (API) from the Garmin server to a server at Hannover Medical School with the aim of a regular feedback and activity adaptation from the supervisor. Participants were free to contact their supervisor per telephone or e-mail at any time in case of questions.

All participants of the exercise group received nutritional counseling, which provided background information on healthy food choices based on general recommendations issued by the German Society for Nutrition (https://www.dge.de/ernaehrungspraxis/vollwertige-ernaehrung/10-regeln-der-dge/10-guidelines-of-the-german-nutrition-society).

### Statistical Analysis

The primary outcome of our study was the change in the metabolic syndrome severity following a 6-month exercise intervention compared to controls. Based on an earlier study using a similar intervention (33) a sample size of 264 participants was calculated to achieve a significant between-group difference for the primary outcome with 90% power and significance level of 0.05 (MedCalc Statistical Software version 17.6, Ostend, Belgium). With an anticipated dropout rate of 18% we calculated a final sample size of 312 subjects for inclusion in the study. For the analysis of the primary and all secondary outcomes, an analysis of covariance model (ANCOVA) was used with the change in the parameter of interest (6 months-baseline) as the response variable. Explanatory variables were gender, the respective parameter at baseline, and the study group (exercise- vs. control). Normality distribution was tested using the Kolmogorov-Smirnov-Test. For all outcomes the analysis was carried out according to the intention-to-treat (ITT) principle, including all randomized subjects. Missing values were replaced by the baseline observation carried-forward method. For descriptive analysis absolute frequencies were calculated for categorical variables and mean and standard deviation (SD) for continuous variables. To test for within-group differences from baseline to end of intervention, a two-sided *Students* T-Test for paired samples was used. Univariate associations between parameters were tested using *Pearson’s* correlation coefficient.

For the current secondary analysis, participants were stratified according to subgroups of normal versus mild to severe depression- or anxiety severity. Stratification was based on results from the baseline HADS questionnaire with scores from 0–7 points set as normal and scores from 8–21 as mild to severe. To analyze whether exercise versus control group changes over time were different for depression and anxiety subgroups, we used the interaction term: time (baseline-6 months) × study group (exercise or control) × subgroup (normal scores or mild to moderate scores) as calculated with the ANCOVA model. The type-I-error was set to 5% (two-sided). All statistical analyses were performed with IBM SPSS 25 Statistics (IBM Corporation, NY, US).

## Results

Of 314 randomized subjects, 274 (87%) completed the intervention, with 28 subjects in the exercise group and 12 in the control dropped out during the 6-month period. We documented no serious adverse event during the intervention in both groups. Subjects in the exercise and control group did not differ for gender distribution, age, body composition, daily physical activity, exercise capacity, and mental health at baseline (**Table 1**).

**Table 1:** Subject characteristics at baseline.

	Exercise group	Control group	p-value
Subjects (women/men)	160 (24/136)	154 (21/133)	p=0.730
Age (years)	48.3 ± 7.9	47.8 ± 8.5	p=0.624
Body weight (kg)	107.6 ± 18.3	106.1 ± 20,3	p=0.425
Body mass index (kg/m2)	33.6 ± 5.3	33.0 ± 5.4	p=0.281
Body fat (%)	33.0 ± 8.4	32.1 ± 8.0	p=0.317
Fat Free Mass (kg)	71.0 ± 11.4	71.3 ± 12.8	p=0.961
Systolic blood pressure (mmHg)	138 ± 13	137 ± 14	p=0.715
Diastolic blood pressure (mmHg)	88 ± 9	88 ± 9	p=0.341
HbA1c (%)	5.7 ± 1.0	5.6 ± 0.9	p=0.301
Total cholesterol (mg/dl)	215 ± 46	214 ± 46	p=0.839
LDL cholesterol (mg/dl)	138 ± 39	137 ± 40	p=0.886
Total physical activity (MET-h/wk)	25.9 ± 23.8	21.8 ± 16.5	p=0.966
Exercise activity (MET-h/wk)	6.5 ± 13.7	6.4 ± 9.0	p=0.081
Exercise capacity (peak watt)	174 ± 36	176 ± 37	p=0.906
Depression severity (points)	5.3 ± 3.2	5.0 ± 3.3	p=0.455
Anxiety severity (points)	4.6 ± 2.6	4.6 ± 3.1	p=0.215

MET, metabolic equivalent of task; differences between groups were analyzed with Students T-Test for unpaired samples or the chi-square test, data are mean ± SD.

### Adherence to Physical Activity, Exercise Training Characteristics, and Exercise Capacity

Questionnaire-estimated exercise activities increased more for subjects in the exercise group during the 6-month intervention (EG pre: 6.5 ± 13.7; post 15.9 ± 20.1 MET-hours/wk; CG pre: 6.4 ± 9.0, post: 10.5 ± 15.6 MET-hours/wk) (p < 0.01 compared to baseline for both groups, p < 0.01 between groups over time). As assessed by the activity monitor worn by the EG subjects, adherence to the scheduled target of 150 min of physical activities per week was 147 ± 46 min/wk. Average steps during the intervention was 9612 ± 2498 steps/day. The maximum power output during incremental exercise testing increased for the exercise- (pre: 1.66 ± 0.38, post: 1.89 ± 0.46 W/kg BW, p < 0.01) and the control group (pre: 1.69 ± 0.40, post: 1.77 ± 0.44 W/kg BW, p < 0.01) with a significant difference between groups of 0.16 W/kg BW [CI95%: 0.10; 0.21] (p < 0.01).

### Health-Related Quality of Life and Mental Health

**Figure 1:**
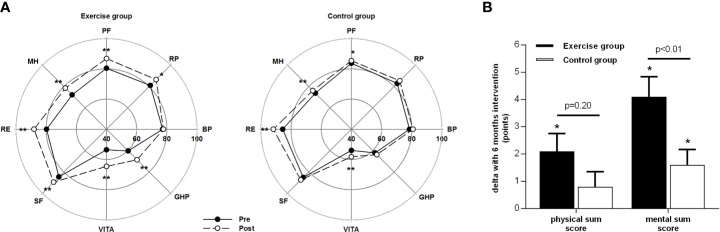
Health-related quality of life as assessed with the SF-36 questionnaire before and after the 6-month intervention. **(A)**: Physical and psychosocial subscales: PF, physical functioning; RP, role physical; BP, bodily pain; GHP, general health perception; VITA, vitality; SF, social functioning; RE, role emotional; MH, mental health. **(B)**: Changes of the physical and mental component sum scores with the 6-month intervention. Data are mean ± SEM, * or ** = significant with p < 0.05 or p < 0.01 respectively from pre to post assessments as analyzed with T-tests for paired samples.

Seven from eight subscales of health-related quality of life (SF-36 questionnaire) increased in the EG and four subscales in the CG from baseline to 6 months (see **Figure 1A**), with significant differences between study groups over time for the subscales 1 (physical functioning), 4 (general health perceptions), 5 (vitality), and 8 (mental health), favoring exercise training. The physical- and mental component sum scores of the SF-36 increased after exercise training, with a significantly greater increase compared to CG for the mental component score (see **Figure 1B**).

Depression severity based on the HADS questionnaire decreased for the EG (–2.0 ± 2.4 points) and the CG (–0.6 ± 2.2 points) (both p<0.01), with a greater decrease over time for subjects in the EG (mean difference –1.4 points [CI95% –1.9; –0.9]; p < 0.01). Anxiety severity decreased for the EG (–1.5 ± 2.4 points) and the CG (–0.7 ± 2.0 points) (both p < 0.01), with a greater decrease over time for subjects in the EG (mean difference –0.9 points [CI95% –1.5; –0.3]; p < 0.01).

### Subgroups of Normal Versus Increased Depression and Anxiety Severity

When stratified for the presence of mild to severe (HADS: 8–21 points) and normal scores (HADS: 0–7 points) of depression and anxiety, the majority of our subjects presented with mental conditions within the normal range of depression and anxiety severity scores (78 and 73%, respectively), and the remaining subjects with mild or moderate severity scores (8–14 points, 22 and 27% for depression and anxiety scores respectively).

**Figure 2:**
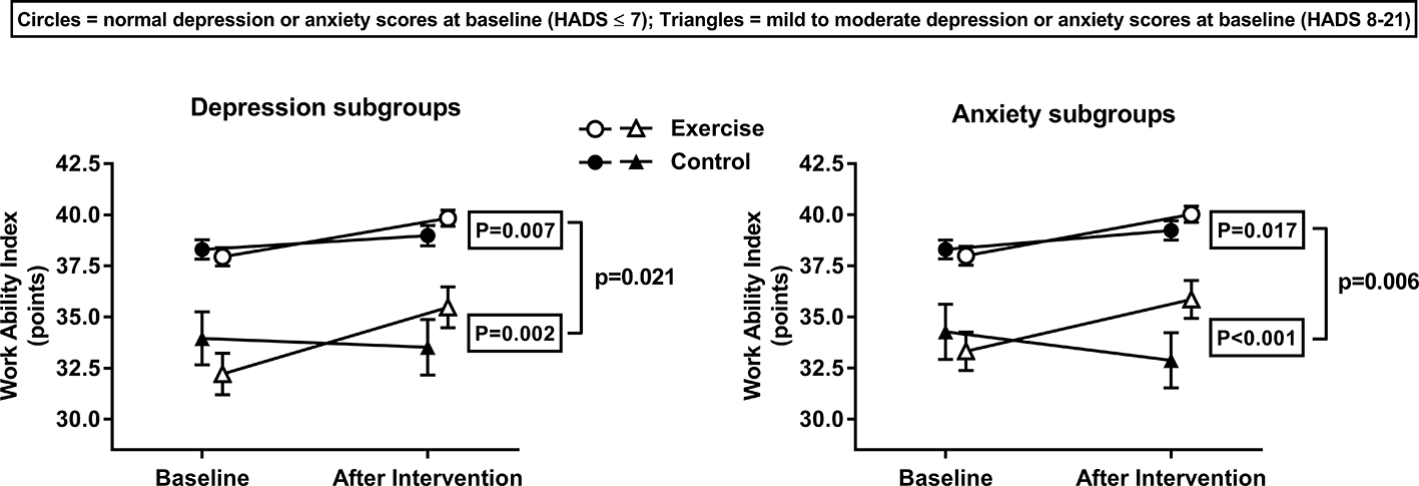
Work ability (total score) as assessed with the work ability questionnaire before and after 6-month exercise training or control. Subjects are stratified according to baseline depression and anxiety severity derived from the HADS questionnaire in subgroups of normal scores (0–7 points, triangles) or mild to moderate scores (8–14 points, circles). Data are mean ± SEM. The framed p-values are given for between-group differences (exercise- versus control group) over time as analyzed with an ANCOVA model. The p-value behind the bracket is given for the interaction of time (baseline-6 months) × study group (exercise or control) × subgroup (normal scores or mild to moderate scores) as analyzed with an ANCOVA model, indicating the 6-month exercise versus control group change in the mild to moderate HADS subgroup is greater than the change in the normal HADS subgroup.

Before starting the intervention, similar values between subgroups were observed for age, body composition, blood lipids, glucose homeostasis, daily physical activity, and cardiorespiratory fitness (**Tables 2** and **3**). In contrast, the total work ability score (**Figure 2**) and sub-items from the work ability index questionnaire (**Tables 2** and **3**), were always lower in the subgroups with increased depression and anxiety severity at baseline (except for item 5 of anxiety, see **Table 3**). For the sub-scales and component sum scores (physical and mental component score) of the SF-36 questionnaire we observed the same pattern at baseline, with lower health-related quality of life for those individuals with increased depression and anxiety severity (**Tables 2** and **3**).

**Table 2:** Measurements before and after the intervention for subjects with normal versus mild to moderate depression scores.

	Subjects without depression							Subjects with mild to moderate depression				
	Whole sub- group baseline	Exercise baseline	Exercise change from baseline	Controls baseline	Controls change from baseline	between groups over time	Whole sub- group baseline	Exercise baseline	Exercise change from baseline	Controls baseline	Controls change from baseline	between groups over time
n (men/ women)	242 (206/36)	129 (109/20)		113 (97/16)			53 (47/6)	29 (26/3)		24 (21/3)		
Age (years)	47.6 ± 8.5	47.9 ± 8.3		47.3 ± 8.8			49.9 ± 6.5	49.9 ± 6.1		49.8 ± 7.1		
Body weight (kg)	106.7 ± 19.6	107.1 ± 19.2	–4.1 ± 4.9**	105.9 ± 20.0	–1.0 ± 4.9*	<0.01	108.9 ± 16.8	109.4 ± 13.6	–4.1 ± 5.3**	108.4 ± 20.4	0.1 ± 5.1	0.01
Body mass index (kg/m2)	33.2 ± 5.5	33.6 ± 5.5	–1.5 ± 3.2**	32.8 ± 5.4	–0.3 ± 1.4*	<0.01	33.8 ± 5.3	33.4 ± 4.6	–1.2 ± 1.5**	34.3 ± 6.1	0.0 ± 1.6	<0.01
Body fat (%)	32.3 ± 8.4	33.1 ± 8.5	–2.2 ± 3.2**	31.5 ± 8.4	–0.3 ± 3.3	<0.01	33.8 ± 6.8	32.7 ± 7.3	–1.5 ± 2.8**	35.1 ± 6.0	–0.1 ± 6.0	0.44
Fat free mass (kg)	71.4 ± 12.3	36.3 ± 13.5	–3.7 ± 4.7**	34.2 ± 13.3	–0.6 ± 4.7	0.20	71.5 ± 9.2	36.9 ± 12.4	–3.0 ± 4.1**	39.0 ± 12.5	–0.3 ± 5.9	0.33
Fasting glucose (mmol/l)	6.1 ± 1.1	6.1 ± 1.3	–0.4 ± 0.8**	6.0 ± 1.0	0.0 ± 0.5	<0.01	6.3 ± 1.1	6.3 ± 1.1	–0.2 ± 0.5*	6.3 ± 1.0	–0.1 ± 0.9	0.55
HbA1c (%)	5.6 ± 0.8	5.7 ± 1.0	–0.2 ± 0.5**	5.5 ± 0.6	–0.1 ± 0.4	0.02	**5.9 ± 1.4**	5.6 ± 1.0	–0.3 ± 1.3	6.0 ± 1.6	–0.3 ± 0.9	0.79
Triglycerides (mmol/l)	2.3 ± 2.0	2.1 ± 1.5	–0.3 ± 1.0**	2.1 ± 1.3	–0.0 ± 0.8	<0.01	2.3 ± 1.6	2.2 ± 1.6	–0.2 ± 0.9	2.3 ± 1.3	–0.1 ± 0.7	0.29
Total cholesterol (mmol/l)	213.2 ± 45.9	215.6 ± 48.1	–9.1 ± 25.0**	211.3 ± 44.0	–1.0 ± 27.0	0.03	215.3 ± 40.7	215.0 ± 37.0	–3.8 ± 20.6	215.8 ± 45.6	–4.2 ± 24.2	0.88
Exercise activity (MET-h/wk)	6.7 ± 12.7	6.8 ± 15.2	10.2 ± 24.2**	6.7 ± 9.3	5.4 ± 17.7**	0.05	5.6 ± 7.4	5.6 ± 6.6	5.3 ± 14.5**	5.9 ± 8.4	0.1 ± 8.6	0.14
Exercise capacity (peak watt)	176.4 ± 37.1	174.6 ± 36.1	15.7 ± 20.0**	177.6 ± 38.0	7.9 ± 21.0**	<0.01	170.3 ± 33.1	175.9 ± 32.6	17.9 ± 21.9	163.5 ± 33.1	3.6 ± 19.8	0.02
MetS-z-Score (arbitrary units)	0.93 ± 0.63	0.92 ± 0.64	–0.29 ± 0.47**	0.93 ± 0.59	–0.05 ± 0.38	<0.01	1.04 ± 0.57	0.96 ± 0.64	–0.27 ± 0.44**	1.07 ± 0.45	–0.09 ± 0.41	0.16
**Work ability (points)**												
Current Work Ability	7.62 ± 1.53	7.66 ± 1.48	0.39 ± 1.27**	7.59 ± 1.59	0.36 ± 1.82	0.04	**6.71 ± 1.75**	6.66 ± 1.59	0.72 ± 1.39**	6.78 ± 1.98	–0.35 ± 1.30	<0.01
Work Ability In Relation To Demands	8.20 ± 1.22	8.21 ± 1.26	0.31 ± 1.10**	8.18 ± 1.18	0.12 ± 1.01	0.11	**7.14 ± 1.31**	7.00 ± 1.22	0.43 ± 1.51	7.30 ± 1.43	–0.22 ± 1.19	0.15
Number of Current Diseases	3.41 ± 1.92	3.39 ± 2.06	–1.15 ± 3.49**	3.45 ± 1.76	–1.26 ± 3.03**	0.93	**2.65 ± 1.76**	2.31 ± 1.95	0.83 ± 3.81	3.09 ± 1.41	0.09 ± 3.26	0.17
Work Impairment Due To Diseases	5.41 ± 0.77	5.39 ± 0.77	–1.80 ± 1.94**	5.44 ± 0.77	–1.74 ± 1.89**	0.79	**5.04 ± 0.84**	4.93 ± 0.84	–2.00 ± 2.17**	5.17 ± 0.83	–2.44 ± 2.13**	0.81
Sick Leaving Last Year	3.82 ± 1.00	3.73 ± 1.07	0.27 ± 1.03**	3.93 ± 0.91	0.05 ± 0.96	0.33	**3.40 ± 1.11**	3.38 ± 1.08	0.10 ± 1.21	3.43 ± 1.16	–0.04 ± 1.11	0.72
Own Prognosis Of Work Ability	6.61 ± 1.07	6.62 ± 1.02	0.13 ± 1.12	6.67 ± 1.05	–0.03 ± 1.03	0.06	**5.67 ± 1.72**	5.66 ± 1.72	0.62 ± 1.68	5.70 ± 1.77	0.39 ± 1.37	0.30
Mental Resources	3.00 ± 0.65	2.95 ± 0.60	0.33 ± 0.67**	3.05 ± 0.70	0.14 ± 0.71*	0.07	**2.37 ± 0.69**	2.28 ± 0.65	0.35 ± 0.61**	2.48 ± 0.73	0.09 ± 0.73	0.36
**Health-related quality of life (points)**												
Physical Functioning	84.3 ± 13.9	82.3 ± 15.0	6.3 ± 11.7**	86.6 ± 12.3	1.4 ± 8.5	0.01	**73.2 ± 19.2**	73.4 ± 18.9	4.5 ± 13.2	72.9 ± 20.0	3.3 ± 16.9	0.73
Physical Role	84.9 ± 26.7	84.1 ± 27.2	3.3 ± 27.2	84.9 ± 26.8	2.9 ± 29.3	0.99	**70.3 ± 32.9**	67.2 ± 31.4	13.8 ± 34.5	74.0 ± 35.0	8.3 ± 43.4	0.98
Bodily Pain	79.8 ± 21.5	78.7 ± 20.9	0.7 ± 17.8	80.4 ± 22.5	2.4 ± 18.8	0.22	**69.1 ± 24.2**	70.0 ± 23.7	–0.3 ± 21.1	68.0 ± 25.3	6.2 ± 21.7	0.29
General Health Perceptions	63.7 ± 16.2	62.6 ± 16.3	8.9 ± 13.0**	65.2 ± 15.8	2.0 ± 13.3	<0.01	**49.4 ± 14.3**	49.6 ± 14.5	6.7 ± 15.6*	49.1 ± 14.4	5.9 ± 13.9	0.78
Vitality	57.2 ± 16.4	57.2 ± 16.2	10.0 ± 16.8**	57.2 ± 16.7	3.7 ± 14.0**	<0.01	**38.6 ± 11.7**	37.4 ± 10.5	15.9 ± 15.4**	40.0 ± 13.2	8.5 ± 17.5*	0.16
Social Role Functioning	89.9 ± 16.6	90.2 ± 16.3	3.0 ± 13.8*	89.7 ± 16.6	0.9 ± 14.9	0.12	**62.7 ± 23.9**	59.1 ± 24.3	14.3 ± 18.9**	66.9 ± 23.2	11.5 ± 22.4*	0.98
Emotional Role	88.0 ± 26.0	85.7 ± 29.0	7.3 ± 29.4**	90.0 ± 22.8	4.8 ± 18.5**	0.78	**57.8 ± 42.0**	50.6 ± 40.5	18.4 ± 34.1**	66.6 ± 43.9	13.9 ± 325.4	0.74
Mental Health	77.1 ± 11.9	76.9 ± 12.0	4.8 ± 10.1**	77.8 ± 11.7	1.8 ± 8.1*	0.02	**54.0 ± 16.1**	53.0 ± 14.0	10.6 ± 14.1**	55.3 ± 18.7	5.6 ± 16.9	0.31
Physical component score	49.0 ± 7.2	48.5 ± 7.3	2.1 ± 6.3**	49.7 ± 7.3	0.7 ± 5.6	0.20	**46.7 ± 8.4**	47.8 ± 8.7	1.1 ± 8.7	46.3 ± 8.6	1.7 ± 8.2	0.96
Mental component score	51.8± 7.2	51.7 ± 7.0	3.0 ± 6.8**	51.8 ± 7.7	1.3 ± 5.3*	0.03	**38.4 ± 11.1**	37.2 ± 9.5	10.2 ± 9.8**	40.9 ± 12.5	3.9 ± 9.1*	0.05

MET, metabolic equivalent of task, Bold-marked values indicate differences at baseline between subgroups (normal versus mild to severe depression) (p < 0.05) as analyzed with Students T-Test for unpaired samples, *p < 0.05 and **p < 0.01 indicate within-group changes in the exercise or control group from baseline as analysed with Students T-Test for paired samples. Differences between the exercise and control groups over time (time × group) in the respective subgroup were analysed by an ANCOVA model controlled for baseline values and gender. Data are mean ± SD.

**Table 3:** Measurements before and after the intervention for subjects with normal versus mild to moderate anxiety scores.

	Subjects without anxiety						Subjects with mild to moderate anxiety					
	Group baseline	Exercise baseline	Exercise change from baseline	Controls baseline	Controls change from baseline	between groups over time	Group baseline	Exercise baseline	Exercise change from baseline	Controls baseline	Controls change from baseline	between groups over time
n (men/ women)	233 (200/33)	121 (104/17)		112 (96/16)			62 (53/9)	37 (31/6)		25 (22/3)		
Age (years)	47.4 ± 8.5						**50.2 ± 6.9**					
Body weight (kg)	106.9 ± 19.0	107.1 ± 18.5	–4.2 ± 5.1**	106.2 ± 19.6	–0.9 ± 5.1	<0.01	107.9 ± 19.6	108.6 ± 17.9	–4.2 ± 4.7**	106.9 ± 22.3	–0.5 ± 4.0	<0.01
Body mass index (kg/m2)	33.3 ± 5.3	33.5 ± 5.2	–1.5 ± 3.3**	33.0 ± 5.3	–0.3 ± 1.5*	<0.01	33.6 ± 6.0	33.9 ± 5.8	–1.3 ± 1.4**	33.2 ± 6.4	–0.2 ± 1.2	0.03
Body fat (%)	32.5 ± 8.2	33.0 ± 8.2	–2.3 ± 3.2**	32.0 ± 8.3	–0.4 ± 3.6	<0.01	33.0 ± 7.9	33.2 ± 8.7	–1.5 ± 2.7**	32.9 ± 7.0	–0.7 ± 5.1	0.04
Fat free mass (kg)	71.4 ± 12.2	71.0 ± 11.2	–0.2 ± 3.7	71.3 ± 13.0	0.6 ± 6.3	0.15	71.6 ± 10.2	71.0 ± 9.4	–1.4 ± 2.6**	71.6 ± 10.8	0.9 ± 4.7	0.69
Fasting glucose (mmol/l)	6.1 ± 1.2	6.1 ± 1.3	–0.4 ± 0.8**	6.1 ± 1.0	–0.1 ± 0.6	<0.01	6.1 ± 1.0	6.1 ± 1.1	–0.2 ± 0.4**	6.0 ± 0.7	0.1 ± 0.6	0.06
HbA1c (%)	5.6 ± 0.8	5.7 ± 1.0	–0.2 ± 0.5**	5.5 ± 0.7	–0.1 ± 0.4	0.10	5.8 ± 1.3	5.7 ± 1.2	–0.3 ± 1.1	5.8 ± 1.5	–0.2 ± 0.9	0.36
Triglycerides (mmol/l)	2.3 ± 1.6	2.2 ± 1.5	–0.3 ± 1.0**	2.1 ± 1.3	–0.02 ± 0.73	0.01	2.5 ± 2.8	2.0 ± 1.6	–0.3 ± 1.0	2.2 ± 1.2	–0.1 ± 0.8	0.19
Total cholesterol (mmol/l)	213.0 ± 45.6	216.3 ± 47.4	–8.5 ± 25.0**	209.9 ± 43.9	–0.4 ± 26.0	0.30	215.7 ± 43.2	213.1 ± 42.5	–6.6 ± 21.8	222.1 ± 44.9	–7.0 ± 28.3	0.88
Exercise activity (MET-h/wk)	6.4 ± 9.8	5.5 ± 10.1	11.3 ± 22.2**	7.5 ± 9.6	4.7 ± 17.9**	0.04	7.0 ± 17.7	10.1 ± 22.3	2.8 ± 23.6	2.8 ± 5.7	3.3 ± 8.6	0.09
Exercise capacity (peak watt)	176.4 ± 34.7	175.6 ± 32.4	14.8 ± 18.8**	176.4 ± 36.9	7.5 ± 21.7**	<0.01	171.1 ± 42.2	172.4 ± 44.3	20.5 ± 24.4**	169.2 ± 39.8	5.6 ± 16.4	<0.01
MetS-z-Score (arbitrary units)	0.97 ± 0.60	0.97 ± 0.62	–0.32 ± 0.47**	0.97 ± 0.58	–0.05 ± 0.38	<0.01	0.90 ± 0.77	0.83 ± 0.68	–0.20 ± 0.42**	0.93 ± 0.56	–0.08 ± 0.41	0.16
**Work ability (points)**												
Current Work Ability	7.61 ± 1.58	7.64 ± 1.53	0.43 ± 1.27**	7.60 ± 1.62	0.05 ± 1.86	0.03	**6.92 ± 1.62**	6.95 ± 1.49	0.51 ± 1.39	6.84 ± 1.84	–0.36 ± 1.11	0.01
Work Ability In Relation To Demands	8.23 ± 1.62	8.23 ± 1.25	0.29 ± 1.07**	8.25 ± 1.12	0.16 ± 1.04	0.30	**7.19 ± 1.37**	7.22 ± 1.32	0.46 ± 1.49	7.08 ± 1.44	–0.34 ± 0.99	0.02
Number of Current Diseases	3.43 ± 1.90	3.40 ± 2.07	–1.18 ± 3.52**	3.48 ± 1.72	–1.37 ± 2.98**	0.66	**2.68 ± 1.84**	2.51 ± 1.97	0.49 ± 3.69	3.00 ± 1.63	0.44 ± 3.24	0.02
Work Impairment Due To Diseases	5.44 ± 0.76	5.43 ± 0.76	–1.78 ± 2.03**	5.46 ± 0.75	–1.65 ± 1.91**	0.57	**5.00 ± 0.82**	4.92 ± 0.80	–2.03 ± 1.82**	5.12 ± 0.88	–2.76 ± 1.83**	0.13
Sick Leaving Last Year	3.78 ± 0.98	3.75 ± 1.03	0.29 ± 1.06**	3.81 ± 0.94	0.17 ± 0.92	0.44	3.63 ± 1.18	3.38 ± 1.19	0.05 ± 1.08	4.00 ± 1.12	–0.60 ± 1.00**	0.12
Own Prognosis Of Work Ability	6.63 ± 1.05	6.62 ± 1.02	0.18 ± 1.15	6.66 ± 1.06	–0.03 ± 0.96	0.11	**5.79 ± 1.69**	5.86 ± 1.64	0.32 ± 1.55	5.80 ± 1.73	0.36 ± 1.58	0.99
Mental Resources	3.00 ± 0.66	2.93 ± 0.65	0.33 ± 0.69**	3.06 ± 0.68	0.14 ± 0.70	0.12	**2.49 ± 0.69**	2.49 ± 0.61	0.32 ± 0.53**	2.46 ± 0.78	0.08 ± 0.78	0.09
**Health-related quality of life (points)**												
Physical Functioning	84.4 ± 13.8	82.8 ± 14.4	6.1 ± 11.7**	86.1 ± 13.0	0.4 ± 8.3	<0.01	**74.2 ± 19.1**	73.1 ± 19.3	5.4 ± 13.0*	75.6 ± 19.1	7.4 ± 15.7*	0.44
Physical Role	84.9 ± 26.7	84.7 ± 26.5	3.7 ± 26.2	84.5 ± 27.5	4.5 ± 30.3	0.80	**72.2 ± 32.4**	68.8 ± 32.4	10.4 ± 36.0	76.0 ± 32.7	1.0 ± 39.8	0.51
Bodily Pain	80.1 ± 21.3	79.1 ± 21.1	0.8 ± 18.4	80.5 ± 21.9	2.5 ± 18.2	0.25	**69.5 ± 24.2**	70.4 ± 22.2	–0.6 ± 18.6	68.2 ± 27.6	5.8 ± 23.8	0.28
General Health Perceptions	63.6 ± 16.5	63.0 ± 16.7	8.4 ± 12.5**	64.5 ± 16.2	2.7 ± 13.4*	<0.01	**51.9 ± 14.3**	50.8 ± 13.3	8.5 ± 16.5**	53.5 ± 16.1	2.4 ± 13.6	0.19
Vitality	56.9 ± 16.8	56.8 ± 17.1	10.1 ± 17.4**	57.1 ± 16.6	4.4 ± 15.2*	<0.01	**42.3 ± 13.4**	42.5 ± 12.2	14.3 ± 13.4**	41.2 ± 14.8	5.6 ± 12.8*	0.01
Social Role Functioning	90.9 ± 15.7	92.0 ± 14.7	2.1 ± 12.2	89.9 ± 16.2	2.7 ± 16.4	0.58	**62.9 ± 23.1**	59.5 ± 22.3	15.1 ± 20.5**	67.2 ± 24.2	3.1 ± 19.1	0.06
Emotional Role	89.8 ± 23.3	88.2 ± 25.4	4.9 ± 25.8*	91.2 ± 21.6	5.5 ± 20.0**	0.22	**55.4 ± 42.3**	49.1 ± 41.8	24.1 ± 39.5**	62.7 ± 42.3	10.6 ± 31.6	0.44
Mental Health	77.9 ± 11.3	78.1 ± 10.6	3.9 ± 8.6**	78.2 ± 12.0	2.3 ± 10.0*	0.19	**54.7 ± 15.2**	53.9 ± 14.5	12.4 ± 15.4**	55.7 ± 16.7	3.2 ± 11.3	0.02
Physical component score	49.1 ± 7.2	48.5 ± 7.2	2.3 ± 6.4**	49.4 ± 7.4	0.7 ± 5.4	0.06	**47.0 ± 8.4**	48.1 ± 8.5	0.6 ± 8.0	47.7 ± 8.5	1.6 ± 8.4	0.69
Mental component score	52.2 ± 6.6	52.2 ± 6.3	2.7 ± 6.5**	52.4 ± 7.1	1.7 ± 6.0**	0.20	**39.2 ± 11.2**	38.5 ± 10.2	9.7 ± 9.8**	39.7 ± 11.6	2.2 ± 7.3	<0.01

MET, metabolic equivalent of task, Bold-marked values indicate differences at baseline between subgroups (normal versus mild to severe depression) (p < 0.05) as analyzed with Students T-Test for unpaired samples, *p < 0.05 and **p < 0.01 indicate within-group changes in the exercise or control group from baseline as analysed with Students T-Test for paired samples. Differences between the exercise and control groups over time (time × group) in the respective subgroup were analysed by an ANCOVA model controlled for baseline values and gender. Data are mean ± SD.

After the 6-month intervention, body mass index decreased and exercise capacity increased, irrespective of normal or increased baseline depression and anxiety severity (**Tables 2** and **3**). The total work ability score increased in the EG compared to the CG over time, in both the normal and mild to moderate baseline depression and anxiety subgroups (**Figure 2**, framed p-values). However, this increase in total work ability was significantly greater after exercise training for subjects with increased depression or anxiety scores at baseline compared to those with normal depression or anxiety severity (**Figure 2**, unframed p-value). For the physical component sum score of the SF-36 we observed no improvement of exercise training in neither depression nor anxiety subgroup (**Tables 2** and **3**). In contrast, the mental component sum score improved for subgroups with increased depression and anxiety severity and for the subgroup with normal depression severity at baseline (**Tables 2** and **3**).

## Discussion

We observed that individuals with increased depression or anxiety severity had a lower self-perceived work ability and health-related quality of life, despite comparable age, body mass index, and other traditional cardio-metabolic risk factors. Six-month telemonitoring-supported and individually guided exercise training improved work ability and health-related quality of life in employees with metabolic syndrome, and this effect was strongest for individuals with higher baseline depression severity or anxiety severity scores.

It is well known that patients with major depressive disorders have a more sedentary lifestyle and engage less in physical activity and healthy lifestyle (34–36). This effect is also observed in anxiety, although to a lesser extent (37). Exercise interventions have been effective in reducing symptoms of anxiety and depression, and in preventing anxiety disorder and major depressive disorders (35, 38, 39). A recent meta-analysis from the European Psychiatric Association regarding exercise in severe mental illness came to the conclusions that exercise as a treatment should be integral part of multimodal treatment for these disorders (40). However, studies concerning the effects of exercise interventions on work ability in psychologically burdened, non-clinical samples are scarce. Depression and anxiety are associated with more sick-days at the workplace, earlier retirement, and less productivity compared to individuals without diagnosed depression and anxiety (41–44). Some studies have been published highlighting psychotherapeutic approaches to improve return-to-work in patients with depression and anxiety, either using face-to-face psychotherapy or internet based devices (45, 46). In our study, a preventive exercise approach was used in non-clinical, but psychologically burdened subjects who were diagnosed with metabolic syndrome. In these individuals who are prone to develop apparent cardiovascular and metabolic diseases, evidence is limited concerning the effects of exercise interventions.

Before starting the intervention, we observed no distinct differences in known cardiovascular and metabolic risk factors like age, body composition, blood lipids, fasting glucose, or exercise capacity between subgroups of normal versus mild to moderate depression or anxiety severity. Notwithstanding these similarities, subjects with increased depression and anxiety manifestation at baseline demonstrated lower work ability and health-related quality of life, highlighting the independent contribution of depression and anxiety on productivity-related outcomes in company workers with metabolic syndrome. These results also reveal that evaluating classical disease risk factors does not uncover limitations in an employee’s ability to work. Measuring and relying on cardiovascular and metabolic parameters may therefore underestimate the impact of mental health on productivity at the workplace and associated costs for the employer and the healthcare system.

After the 6-month guided and telemonitoring-supported physical activity promotion, participants improved several anthropometric and cardio-metabolic outcomes, including body weight, blood pressure, fasting glucose, and blood lipids (27). As observed by the current analysis, the total work ability index increased for employees with both normal and increased depression and anxiety severity after exercise training. Noteworthy, the improvement in subgroups with mild to moderate severity scores was greater compared to that observed in subgroups with normal severity scores. This suggests that employees with metabolic syndrome and elevated depression and anxiety scores could most benefit from such interventions, and may be of particular interest for programs aiming at indicated prevention. Since these individuals (presenting both metabolic syndrome and mental disease severity) are at enlarged risk for disease progression, but also productivity-related limitations at the workplace, our study suggests to establish structured and guided lifestyle interventions especially for these employees.

Our study has strengths and limitations. Strengths include the randomized-controlled design with a large number of participants. The use of the HADS questionnaire is a limitation as it presents a rather rough estimation of anxiety and depression symptoms. Our observed results should be tested in further studies using a structured interview to confirm the diagnosis of major depressive disorders or anxiety disorders. Furthermore, we cannot discriminate whether the improvements in quality of life are due to the exercise intervention itself or secondary to the increased social support of participants. We observed weak correlations between changes in exercise capacity and changes in health-related quality of life subscales, but because we did not assess social support, we could not test the influence of this factor.

In conclusion, the present analysis of a randomized-controlled physical activity program for company employees with diagnosed metabolic syndrome reports strongest effects on work ability and overall quality of life for individuals with mild to moderate depression- and anxiety disorders. Of importance, the observed effects were most pronounced for participants with higher depression severity or anxiety severity scores at baseline. Our findings also indicate that a telemonitoring-supported physical activity intervention is feasible to guide and supervise lifestyle changes for a large number of individuals, irrespective of their residence or workplace Our results implicate to offer similar interventions to a broader workplace population, not only to reduce individual disease risk but also to possibly ease the burden in healthcare and employers costs arising from metabolic syndrome and mental disease conditions.

## Data Availability Statement

The datasets generated for this study are available on request to the corresponding author.

## Ethics Statement

The studies involving human participants were reviewed and approved by Ethics Committee of Hannover Medical School. The patients/participants provided their written informed consent to participate in this study.

## Author Contributions

SH, DB, CT, MS, DH-K, AH, and UT planned and designed the study. RE, LN, and DL recruited participants. AK, GP, PB, HS, SR, TS, JE, AAH, KK-V, and RE collected the data. AK, GP, PB, HS, SR, TS, JE, CT, AAH, KK-V, and MK processed the exercise test, anthropometric, body composition, metabolic data, and supervised the intervention. MK processed the dietary intake data. SH and DB calculated the sample size and were responsible for the statistical analyses. SH and KK wrote the first draft of the manuscript. LN, DH-K, AH, MS, and UT contributed to the discussion and reviewed/edited the manuscript. All authors contributed to the article and approved the submitted version.

## Funding

This study was supported and funded by grants from Audi BKK health insurance and the German Research Foundation through the Cluster of Excellence “REBIRTH”. The funder Audi BKK health insurance was not involved in the study design, collection, analysis, interpretation of data, the writing of this article or the decision to submit it for publication.

## Conflict of Interest

RN and LN were employed by company Volkswagen AG, and DL was employed by Audi BKK health insurance.

The remaining authors declare that the research was conducted in the absence of any commercial or financial relationships that could be construed as a potential conflict of interest.
